# Position Statement of the Indian Academy of Medical Genetics on Next Generation Sequencing-Based Testing for Rare Genetic Disorders

**DOI:** 10.1007/s12098-026-06124-w

**Published:** 2026-06-16

**Authors:** Anju Shukla, Sameer Bhatia, Mounika Endrakanti, Deepti Gupta, Amita Moirangthem, Prajnya Ranganath

**Affiliations:** 1 Department of Medical Genetics, Kasturba Medical College, Manipal Academy of Higher Education, Manipal, Karnataka India; 2 Department of Pediatrics, Noida International Institute of Medical Sciences, Greater Noida, 201308 Uttar Pradesh India; 3 Division of Genetics, Department of Pediatrics, All India Institute of Medical Sciences, New Delhi, 110029 India; 4 Institute of Medical Genetics and Genomics, Sir Ganga Ram Hospital, New Delhi, 110060 India; 5 Department of Medical Genetics, Sanjay Gandhi Postgraduate Institute of Medical Sciences, Lucknow, Uttar Pradesh India; 6 Department of Medical Genetics, Nizam’s Institute of Medical Sciences, Hyderabad, 500082 Telangana India

**Keywords:** Rare genetic disorders, Next generation sequencing, Genetic counseling

## Abstract

Next-generation sequencing (NGS)-based tests are being increasingly employed by clinicians for obtaining a genetic diagnosis in individuals and families with possible genetic disorders. However, there is a significant disparity in the genomic knowledge and skills of the clinicians employing these tests for care and management of families with rare genetic disorders. Through the current document, the Society for Indian Academy of Medical Genetics (SIAMG) aims to provide guidance and consideration in terms of the type of tests available, their appropriate applications, and result interpretation in order to make judicious use of these tests for rare disease diagnosis.

## Background and Rationale

Next-generation sequencing (NGS) is a high-throughput DNA sequencing technology that allows rapid sequencing. Through massively parallel sequencing of millions of small DNA fragments, NGS enables the sequencing of each nucleotide multiple times, thereby providing high accuracy of detection of sequence variations and drastically reducing the time taken. NGS is being widely used for rapidly producing vast amounts of genomic data in a cost-effective manner. NGS testing can span either a specific panel/set of genes, exome (exons of all genes), or the entire genome, referred to as panel testing, exome sequencing (ES), or genome sequencing (GS), respectively. The choice of NGS testing is often made based on the clinical or research question.

NGS-based testing has transformed the landscape of genomic diagnostics across the globe. In India, where genetic disorders contribute significantly to pediatric and adult morbidity and mortality, these tests are being increasingly utilised by clinicians for precise molecular diagnosis. A definitive molecular diagnosis is imperative for informed genetic counseling, prognostication, management, surveillance and providing reproductive options to families with genetic disorders.

Though these tests are widely available and indicated in several clinical scenarios, they require a baseline understanding of the genetic basis of diseases for appropriate application and usage. This in turn, leads to rational use of the available resources and results in apt care and counseling for the family. This position statement by the Indian Academy of Medical Genetics seeks to offer a structured, evidence-informed framework for the implementation and responsible use of NGS-based testing. The authors aim to outline key areas ranging from the different types of tests, their applications, interpretation and challenges. By addressing both the promise and the pitfalls of these tests, the authors aim to inform the clinicians utilising these tests for the care of families with rare genetic disorders.

## Current Evidence and Practice Landscape

### Types of NGS-Based Tests and Their Applications

NGS is a high-throughput sequencing technology that can be used to sequence variable amounts of captured target regions across the human genome. The following tests are currently being practiced in varying clinical scenarios [1, 2].

#### Targeted Gene Panels

This test is limited to sequencing of a curated set of genes associated with a specific clinical phenotype, such as non-syndromic hearing loss, cardiomyopathy, or skeletal dysplasia. These panels offer high sequencing depth and copy number variant (CNV) detection, often at a lower cost compared to ES or GS, making them more efficient diagnostic tool for well-characterised and clinically recognisable conditions. By focusing on a limited number of genes, there is a reduction in sequencing costs, computational requirements, and data storage needs while maximising clinical sensitivity. When choosing a panel, one must ensure that the target region is covered completely without missing any clinically relevant variant, whether in the coding or non-coding region of the gene. A comprehensive gene panel can identify CNVs as well and is preferable as it would be able to investigate the complete spectrum of variants for the disorder(s) in question. While gene panels are efficient, negative results may require additional testing with ES or GS [3]. Hence, panel tests have largely been replaced by ES and GS. However, a panel test is still preferable in genetic disorders with clinical heterogeneity, where a broader test is likely to result in a higher number of uncertain and difficult to interpret variants, e.g., inherited cardiomyopathies and familial cancers. In these scenarios, a well-curated panel with inclusion of clinically relevant genes and their variants may remain the test of choice.

#### Exome Sequencing

This test sequences the protein-coding regions known as exons and their boundaries, covering approximately 20,000 genes present in the human genome. As approximately 85% of the disease-causing variations occur in exons, ES serves as a targeted yet comprehensive tool for diagnosing Mendelian disorders. It provides a higher diagnostic yield than gene panels and generates a manageable dataset for interpretation, making it a cost-effective technique. ES has now been established as the most efficient and cost-effective test for the diagnosis of rare Mendelian genetic disorders presenting prenatally or postnatally [4, 5]. However, ES cannot detect most of the noncoding variants, repeat expansions and structural variants. The recent advancements in tools have made the recognition of CNVs feasible to a significant extent. The diagnostic yield from CNV analysis from ES data ranges from 1% to approximately 30% in the postnatal setting and has recently been found to be comparable to that of chromosomal microarray [6, 7]. The CNV calls obtained from the ES should be validated by an appropriate test, which includes chromosomal microarray, qPCR, MLPA, etc. A few other variations of this test are available, which may target the exons of a subset of genes e.g., a Mendeliome (commonly known as clinical exome), which covers the exons of those genes that have validated disease-gene associations or in other words, those genes that are known to be associated with a human disease. This test can be employed for individuals who have a clinically recognizable phenotype, such as neurofibromatosis, Joubert syndrome, mucopolysaccharidoses, etc., where the variant is likely to be found in the subset of genes already known to cause these disorders. The diagnostic yield of ES lies between 30–40% empirically and may vary widely depending on clinical findings. A trio ES is likely to increase the diagnostic yield by 5–15% as compared to a singleton proband-only ES [4].

#### Genome Sequencing

This test sequences over 90% of the genome, resulting in more comprehensive and uniform coverage. It enhances the detection of SNVs, intronic variants, CNVs, structural rearrangements (e.g., translocations, inversions), and repeat expansions. However, read depth is lower than targeted sequencing approaches, potentially limiting the detection of mosaicism. Despite its advantages, bioinformatics tools for GS are still evolving, and the high costs of sequencing and data storage remain challenging. As sequencing technologies advance and costs decrease, GS is expected to become the primary method for comprehensive genetic testing in clinical settings [3, 8]. Various studies have shown an additional 5–10% increment in the yield of diagnosis with the application of GS [4]. However, the current cost and challenges of analysis and interpretation limit its use as the first line of testing in a resource-limited setting [9]. Currently, ES/GS are used as a first tier or following an unremarkable chromosomal microarray test result, especially in disorders of developmental delay with or without malformations [4].

#### Indications for NGS-Based Testing

Though NGS-based tests are being utilised for several applications which involve high throughput sequencing, the current document aims to address the indications of these tests for investigating individuals/families with a clinical possibility of a rare genetic disorder, in particular Mendelian/single gene/monogenic disorders [3]. The clinical presentations of Mendelian disorders are extremely varied and may have a single system or multisystem involvement. The age range of clinical manifestations varies from antenatal to some disorders presenting in the geriatric age group. The possibility of a Mendelian disorder is determined by clinical symptoms fitting into a specific clinically recognisable genetic disorder. However, often the history, pedigree and clinical findings suggest a possibility of an underlying genetic etiology, but without a clinical diagnosis or any differential diagnosis, they are referred to as undiagnosed disorders. The recognition of clinical findings of a possible genetic disorder could be challenging due to the rarity of these disorders and, hence, depends on the genetic expertise of the physician. 

### Indications and Applications of Appropriate NGS-Based Tests for Diagnosis

- The clinical diagnosis and the underlying genetics of a disorder determine the appropriate NGS-based test selection and are indicated in the following scenarios:
  - ∘ If there is a clinical possibility of a genetic disorder in which sequence variants are the most common or exclusive cause of the condition.
  - ∘ For genetic disorders that show genetic heterogeneity, i.e. the disorder is caused by more than one gene.
  - ∘ If there is a large target region to be sequenced, i.e. those disorders caused by variants in large genes not easily amenable to Sanger sequencing.
- Gene panels may be used where the genetic basis is very well established, and it is unlikely that a causative variant may lie outside the genes/regions covered in the panel.
- ES is now utilised as the first-tier test for disorders which are caused by SNVs and indels.
- CNVs detected from ES/GS should be validated by another method before clinical use.
- ES or GS can be employed for undiagnosed disorders with a high likelihood of genetic etiology.

### Special Considerations for NGS-Based Testing for Prenatal Diagnosis

Detection of genetic etiology for fetal structural abnormalities warrants the use of genetic testing in utero. Traditionally, fetal karyotype and a few targeted tests were utilized for testing in the antenatal period. Fetal karyotype has now been replaced with chromosomal microarray, which has significantly increased resolution for the detection of chromosomal imbalances. However, fetal structural anomalies suggesting possible monogenic disorders necessitate the use of NGS for establishing a molecular diagnosis. The most common type of NGS test employed for prenatal diagnosis of a fetus with structural abnormalities is ES. The diagnostic yield of ES depends on the case selection and fetal phenotype and ranges from 2–53% in various studies [3, 10]. Trio ES is the recommended line of testing for prenatal diagnosis. A detailed pretest counseling with information regarding utility, diagnostic yield, and detection of uncertain and secondary findings in the ES should be discussed with the family. 

### Key Points

- ES can be used as a first-tier testing when the fetal anomalies suggest a possible monogenic disorder, usually with the opinion of a physician/team with expertise in clinical/medical genetics (e.g., Arthrogryposis, skeletal dysplasia, hydrops fetalis etc.).
- ES may be/ is used when a chromosomal microarray has returned normal results in a fetus with clinical suspicion of a genetic disorder.
- A detailed and informed genetic counseling must precede ES in the prenatal scenario to include the indication of the test, its utility and limitations and the types of results the test may yield.
- The post-test counseling for these scenarios should be provided by an expert/team with sound knowledge of genomics, equipped to handle the complexities of variant interpretation and relevant genetic counseling skills.

## Analysis, Interpretation and Reporting of Genomic Variants

**Fig. 1:**
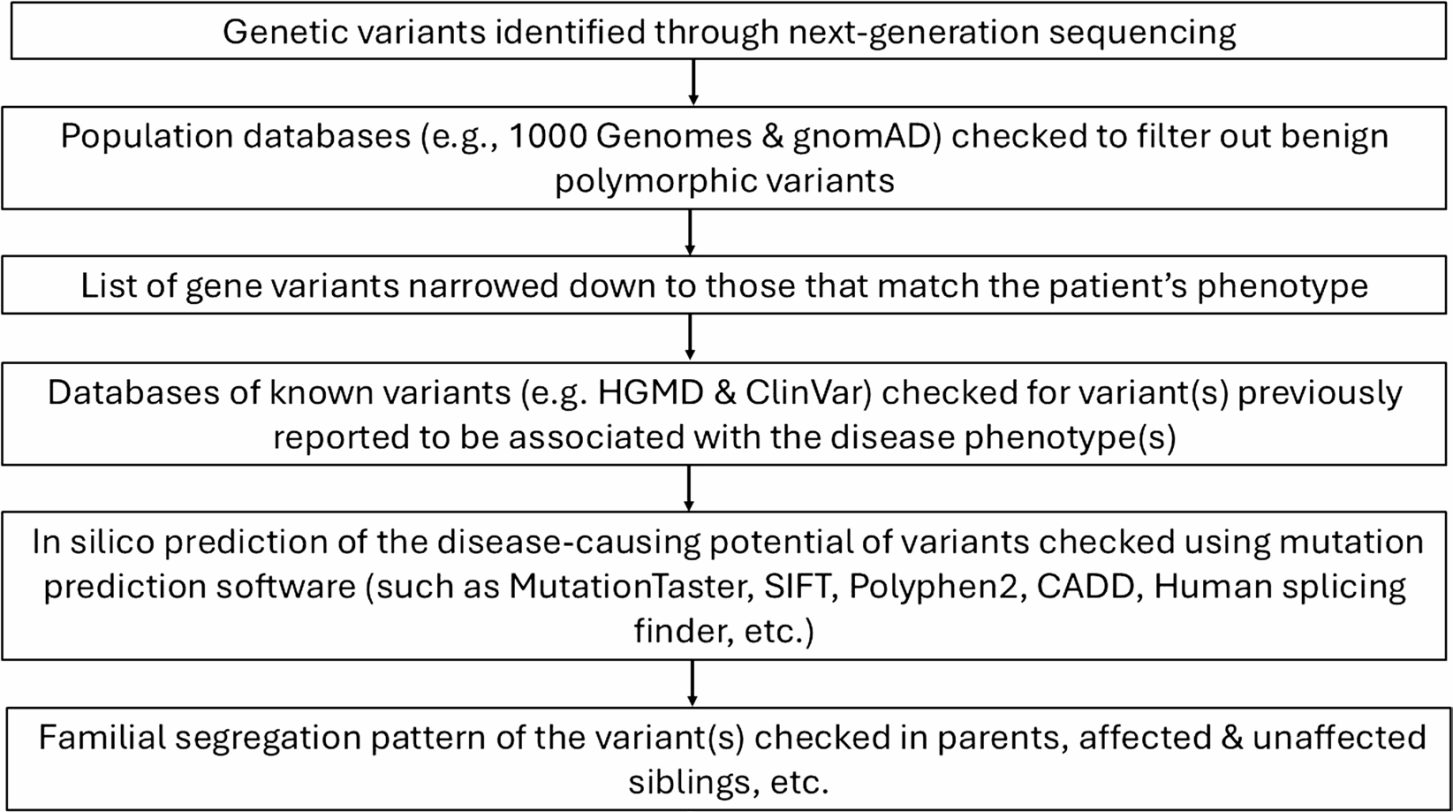
Flowchart showing the step-wise interpretation of variants detected through next-generation sequencing. URLs of the databases mentioned in the figure are as follows: 1000 Genomes: https://www.internationalgenome.org/1000-genomes-browsers/index.html gnomAD: https://gnomad.broadinstitute.org/ HGMD: https://www.hgmd.cf.ac.uk/ac/index.php ClinVar: https://www.ncbi.nlm.nih.gov/clinvar/ MutationTaster: https://www.mutationtaster.org/ SIFT: https://sift.bii.a-star.edu.sg/ Polyphen2: https://genetics.bwh.harvard.edu/pph2/ CADD: https://cadd.gs.washington.edu/ Human splicing finder: https://bio.tools/human_splicing_finder

The genomic sequences that are obtained through NGS of the test sample are aligned and compared to the corresponding reference genomic sequences. The differences between the test sequence and the reference sequence are called variants. NGS-based tests sequence large target areas of the genome, and a huge number of genetic variants are identified. The number of genetic variants detected through ES can range from ~20,000 to ~500,000, while GS may detect around 2 to 5 million variants. The majority of variants identified through broad-spectrum NGS-based testing platforms are benign polymorphic variants that are present in the normal population. In most cases, only one or two variants would be causally related to the disease. Analysis of the huge amount of data generated through NGS testing platforms to sort through the large number of variants and identify the actual disease-causing variant(s) requires expertise and use of bioinformatics tools. The variant data is analyzed through an algorithm based on phenotypic match, databases of known polymorphic variants and pathogenic variants, mutation prediction software results, familial segregation pattern, etc., to sort and filter out the exact disease-causing variant(s) from the other incidentally detected thousands of variants, as shown in Fig. 1. Detailed clinical phenotyping is an important prerequisite because phenotypic match is one of the most important parameters taken into consideration when sifting through the large number of identified variants. The American College of Medical Genetics and Genomics and the Association for Molecular Pathology (ACMG/AMP) have formulated guidelines for the classification of gene variants, using criteria based on population data, computational data, functional data, and segregation data. Based on these guidelines, variants are categorized as ‘pathogenic’, ‘likely pathogenic’, ‘uncertain significance’, ‘likely benign’, and ‘benign’ [11]. Further refinement of these guidelines is available through the ClinGen resource (https://clinicalgenome.org).

Usually, in the clinical setting, identification of a pathogenic or likely pathogenic gene variant(s) that match the clinical phenotype and follow the expected familial segregation pattern is confirmatory for the diagnosis of the genetic disorder associated with that gene. Benign and likely benign variants are usually not of clinical significance. Variants classified as ‘variants of uncertain significance’ (VUS) are those variants whose clinical relevance remains unknown from the current evidence of pathogenicity [12]. As variant databases get updated and scientific literature builds up, many VUS get reclassified as either benign or pathogenic over time. Therefore, patients found to have VUS should be kept under regular follow-up, and the variant status should be rechecked at periodic intervals of 6 mo to a year. Alternatively, further analysis of these variants through functional characterisation studies using cell lines and/or model systems can be used to investigate whether they are disease-causing or not. Also, re-analysis should be periodically carried out for individuals in whom the test did not identify any clinically relevant variant.

Another category of genetic variants that deserves mention is the secondary findings (SF). These variants are intentionally sought in specific genes recommended by the American College of Medical Genetics (ACMG) [initially 53 genes and now updated to 84 genes in ACMG SF (current version v3.3)] [13, 14]. It is recommended to report pathogenic and likely pathogenic (P/LP) variants present in these genes associated with medically actionable conditions such as hereditary cancers, cardiovascular diseases and inborn errors of metabolism. Surveillance with appropriate intervention for the specific SF is of relevance for the patient and at-risk family members. Pre- and post-test counseling hold special significance when reporting SFs. In pre-test counseling, patients should be informed of what SFs are, and the possible SF outcomes and their clinical significance, if identified. Every lab/centre should determine its policy for reporting these variants, depending on its infrastructure and capability to handle these variants. 

### Key Points

- Interpretation of variants should follow ACMG-AMP guidelines, using standardized criteria and multidisciplinary input.
- VUS should not be used for clinical decision-making without supporting evidence and should be periodically reviewed.
- Familial segregation, phenotypic correlation and functional data enhance confidence in variant interpretation.
- The physician ordering the test must be familiar with the possibility of secondary findings and discuss the same with patients/families undergoing NGS based testing.

## Challenges and Limitations of NGS-Based Tests

Notwithstanding its monumental impact in genomics, the NGS revolution has not been without challenges, some of which persist. They can be related to the technique itself or often to the downstream bioinformatic analysis and variant prioritization. A diagnostic laboratory providing genomics services must validate every assay developed and maintain set quality assurance parameters. Though technical standard guidelines are available [15], implementation is not uniform in India at present. Certain regions of the genome, which are complex, may lack adequate coverage and are prone to errors, leading to false calling of variants. Low level of mosaicism may remain undetected especially when the sequencing is not of optimal depth. The vast quantity of data generated by NGS necessitates complex bioinformatics algorithms to process and analyze. Other challenges include data storage, trained manpower to analyze these data and setting up efficient pipelines of variant prioritization, including the complex analysis of genomic structural variations like translocations, inversions, mobile insertion elements and repeat expansions. Also, databases available for population-specific genomic variations of the Indian population are limited. The relatively high cost of NGS based tests has often been cited as a deterrent to its wider application, which however, continues to decline. A significant challenge that is expected to remain is the resolution of VUS. 

### Key Points

- It is essential to acknowledge the challenges and limitations of the NGS-based testing when using it for patient care.
- A few classes of disorders, which are caused by complex genetic variants like imprinting and repeat disorders, amongst others, may require specialized tests and would be out of scope of the above mentioned NGS based tests.
- Challenges related to the identification of most genetic variants using a single test are likely to be addressed with improved technologies like long-read sequencing, which has high sensitivity and specificity for repeat expansions and structural variants.
- Labs with high technical standards and trained manpower is the need of the hour for appropriate variant interpretation.
- Increasing the availability of population-specific variants is also likely to improve variant interpretation.

## Referral to Genetic Services

It is imperative that any individual and/or family with a clinical possibility of a genetic disorder should be evaluated with a thorough clinical history, physical examination and appropriate investigations preceding NGS testing. As these disorders are individually rare and present diagnostic challenges to many medical practitioners, an appropriate and timely referral to a trained medical geneticist for evaluation is recommended. A decision to order and select NGS-based tests should preferably be made by a geneticist or in consultation with a geneticist. The need for detailed phenotyping, counseling regarding the complexities of NGS-based tests and results mandates the involvement of a trained geneticist or a qualified genetic counsellor. 

### Key Points

- Clinical red flags such as syndromic features, consanguinity, or familial disorders warrant early referral to a geneticist.
- Geneticists provide critical input in test selection, result interpretation, and patient counseling.
- Early referral improves diagnostic yield, reduces healthcare costs, and avoids unnecessary investigations.
- A collaborative approach involving primary clinicians and geneticists ensures optimum patient-centred care.

## Conclusions

Next-generation sequencing has significantly broadened the scope of genetic testing, offering various approaches, including targeted gene panels, exome and genome sequencing, each suited for different clinical and research applications. Also, data has emerged which shows that use of NGS earlier in the diagnostic algorithm is more cost-effective when the long diagnostic odyssey and various empiric treatment procedures the patient undergoes are considered [16]. The accessibility of these tests has significantly improved in the last decade. However, several challenges in the application of these tests remain, including genomic literacy of physicians, competent genomic labs, trained manpower, interpretation of results, in particular the variants of uncertain significance and secondary findings and finally the expertise to handle the complex pre- and post-test genetic counseling issues. The present document is meant to provide guidance to health care professionals applying these tests for the investigation of rare genetic disorders in their practice so that they can be applied effectively for improved patient outcomes.
