# Correction to: Position Statement of the Indian Academy of Medical Genetics on Next Generation Sequencing-Based Testing for Rare Genetic Disorders

**DOI:** 10.1007/s12098-026-06333-3

**Published:** 2026-07-13

**Authors:** Anju Shukla, Sameer Bhatia, Mounika Endrakanti, Deepti Gupta, Amita Moirangthem, Prajnya Ranganath

**Affiliations:** 1 Department of Medical Genetics, Kasturba Medical College, Manipal Academy of Higher Education, Manipal, Karnataka India; 2 Department of Pediatrics, Noida International Institute of Medical Sciences, Greater Noida, 201308 Uttar Pradesh India; 3 Division of Genetics, Department of Pediatrics, All India Institute of Medical Sciences, New Delhi, 110029 India; 4 Institute of Medical Genetics and Genomics, Sir Ganga Ram Hospital, New Delhi, 110060 India; 5 Department of Medical Genetics, Sanjay Gandhi Postgraduate Institute of Medical Sciences, Lucknow, Uttar Pradesh India; 6 Department of Medical Genetics, Nizam’s Institute of Medical Sciences, Hyderabad, 500082 Telangana India

**Correction to: Indian Journal of Pediatrics**

10.1007/s12098-026-06124-w

Due to a technical tagging error in the XML, the individual author names under the author group "Executive Committee of the Society for Indian Academy of Medical Genetics (SIAMG)" were omitted from the online version on SpringerLink. 

**Published Form (with error):**

The members of the author group "Executive Committee of the Society for Indian Academy of Medical Genetics (SIAMG)" were not individually listed or tagged. 

**Corrected Form:**

The individual names under the "Executive Committee of the Society for Indian Academy of Medical Genetics (SIAMG)" should be included and tagged as follows: Anju Shukla, Sameer Bhatia, Mounika Endrakanti, Deepti Gupta, Amita Moirangthem, Prajnya Ranganath, Neerja Gupta, Ratna Dua Puri & Madhulika Kabra.

The original article has been corrected.

